# In Vitro and In Vivo Effects of a Copper(II)-Hydrazone Complex Against Human Osteosarcoma

**DOI:** 10.3390/pharmaceutics18030372

**Published:** 2026-03-17

**Authors:** Lucía Santa Maria de la Parra, Matías H. Assandri, Luisina M. Solernó, María de los A. Serradell, Daniel F. Alonso, Juan Garona, Lucía M. Balsa, Ignacio E. León

**Affiliations:** 1 Centro de Química Inorgánica (CEQUINOR CCT-CONICET La Plata, Asociado a CIC), Departamento de Química, Facultad de Ciencias Exactas, Universidad Nacional de La Plata (UNLP), La Plata B1900, Buenos Aires, Argentina; luciasantamaria@quimica.unlp.edu.ar (L.S.M.d.l.P.);; 2 Cátedra de Microbiología, Departamento de Ciencias Biológicas, Facultad de Ciencias Exactas, Universidad Nacional de La Plata (UNLP), La Plata B1900, Buenos Aires, Argentina; massandri@exactas.unlp.edu.ar (M.H.A.); maserr@biol.unlp.edu.ar (M.d.l.A.S.); 3 Centro de Oncología Molecular y Traslacional (COMTra), Universidad Nacional de Quilmes, Bernal B1876, Buenos Aires, Argentina; lusolerno@gmail.com (L.M.S.); danielfalonso@gmail.com (D.F.A.);; 4 Unidad de Investigación Biomédica en Cáncer (IBioCAN), Centro de Medicina Traslacional, Hospital de Alta Complejidad en Red El Cruce “Dr. Néstor Carlos Kirchner” S.A.M.I.C, Florencio Varela B1888, Buenos Aires, Argentina; 5 Instituto de Ciencias de la Salud, Universidad Nacional Arturo Jauretche (UNAJ), Florencio Varela B1888, Buenos Aires, Argentina; 6 Cátedra de Fisiopatología, Departamento de Ciencias Biológicas, Facultad de Ciencias Exactas, Universidad Nacional de La Plata (UNLP), La Plata B1900, Buenos Aires, Argentina

**Keywords:** metallodrug, copper, osteosarcoma, preclinical studies

## Abstract

**Introduction:** Osteosarcoma (OS) is the most common primary malignant bone tumor in children and young adults, with poor prognosis due to relapse, metastasis, and chemoresistance. The search for novel metal-based therapeutics has highlighted copper complexes as promising candidates. Here, we report the in vitro and in vivo antitumor activity of a tetranuclear Cu(II)-hydrazone complex (Cu_4_L_4_) derived from (E)-5-chloro-N′-(2-hydroxy-3-methoxybenzylidene)thiophene-2-carbohydrazide. **Results:** Cytotoxic assays on MG-63 OS cells revealed potent activity with an IC_50_ of 0.50 ± 0.04 µM, significantly surpassing its free ligand (IC_50_ = 13.9 ± 1.6 µM) and cisplatin (IC_50_ = 39.0 ± 1.8 µM). This tetranuclear complex outperforms mononuclear Cu-hydrazones analogs (e.g., 4-fold vs. CuHL1, 2-fold vs. CuHL2, 5-fold vs. CuHL3, 17-fold vs. CuHL4,), and Cu_4_L_4_ also exhibits reduced clonogenic survival, induces reactive oxygen species production, and promotes late apoptosis as a main mechanism, being the main mechanism of action involved in anticancer activity. In multicellular tumor spheroids, the complex maintained strong cytotoxicity (IC_50_ = 4.11 ± 0.12 µM), impaired spheroid integrity, and markedly inhibited cell migration at sub-IC_50_ concentrations. The tetranuclear architecture confers markedly enhanced antitumor activity relative to the corresponding mononuclear Cu–hydrazone complexes (e.g., 2-fold vs. CuHL1, 4-fold vs. CuHL2, 2-fold vs. CuHL3). In a xenograft model, sustained administration of Cu_4_L_4_ (2 mg/kg, i.p., twice weekly) inhibited tumor growth by 43.6%, reduced mitotic index, and increased necrotic area without significant systemic toxicity. **Conclusions:** Overall, Cu_4_L_4_ displayed potent and selective antitumor activity against OS cells in 2D, 3D, and in vivo models, underscoring copper–hydrazone complexes as promising scaffolds for the development of new therapies against OS.

## 1. Introduction

Cancer remains one of the leading causes of mortality worldwide [1]. Among the various malignancies, osteosarcoma (OS) stands out as the most frequent primary bone cancer in children and young adults, and is characterized as a high-grade tumor [2]. A hallmark of OS is the production of immature osteoid matrix by rapidly dividing and highly invasive cells.

The current therapeutic management of OS combines surgical resection with pre- and postoperative chemotherapy. Standard regimens typically include methotrexate, doxorubicin, and CDDP, with ifosfamide or etoposide also being administered in some cases [3,4]. Despite such multimodal approaches, nearly 40% of patients still experience local relapse or distant metastasis, which contributes to the high mortality rate of OS. Furthermore, survival outcomes remain highly unequal: while high-income countries report 5-year survival rates of up to 70%, these values drop to approximately 45% in low- and middle-income regions [5].

Given that both therapeutic progress and patient survival have stagnated, there is an urgent need to develop new therapeutic strategies for OS. In this regard, metallodrugs have emerged as a relevant class of anticancer agents with applications in breast, colorectal, lung, and bone cancers [6,7]. Platinum-based compounds, particularly cisplatin (CDDP), carboplatin, and oxaliplatin, are among the most widely used chemotherapeutics [8]. However, intrinsic and acquired resistance and side effects of platinum drugs significantly limits their long-term efficacy [9]. Consequently, research has turned toward non-platinum metal-based complexes—including ruthenium, vanadium, and copper derivatives—as promising alternatives [10,11,12].

In particular, copper-based compounds have demonstrated notable antitumoral activity in vitro and in vivo across a variety of tumor models [13,14,15,16,17,18,19]. Their proposed mechanisms of action involve the induction of reactive oxygen species (ROS) and subsequent DNA damage, as well as proteasome inhibition and the targeting of cancer stem cells [20,21]. Importantly, several copper complexes have shown stronger antitumor activity than their free ligands, underscoring the critical role of the metal center in mediating these effects [22,23,24]. Nevertheless, only a limited number of copper complexes have been reported to exert significant activity against OS cells [25,26,27,28].

**Figure 1:**
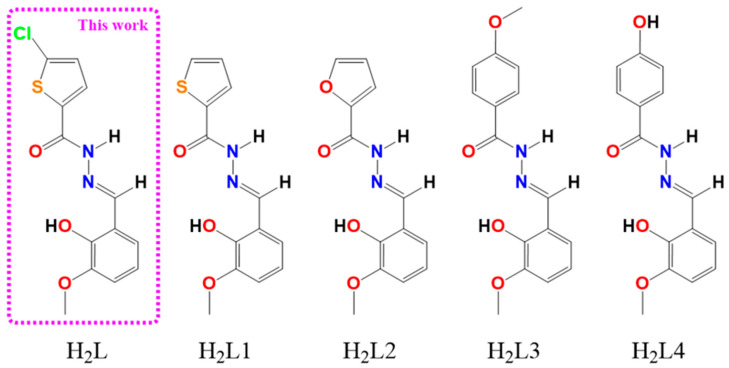
Schematic structures of ligand H_2_L and similar ligands reported previously by our group.

In recent years, hydrazones and their metal complexes gained the attention of researchers due to their possible pharmacological properties, particularly as anticancer therapy [29]. Because the NH_2_ group is inactivated during the condensation reaction of active hydrazides with certain hydroxyaldehydes, stable hydrazones with conserved activity and reduced toxicity are produced [30]. Our group has previously reported the anticancer activity of some copper(II) complexes with the hydrazone ligands presented in Figure 1, and even proved the high efficacy of one of them in an OS in vivo model [17,27,28,31,32,33].

Considering the high mortality associated with OS and the limitations of current treatments, this study focuses on the evaluation of the in vitro and in vivo antitumor activity of the Cu(II) complex Cu_4_L_4_, derived from the hydrazone ligand (E)-5-chloro-N′-(2-hydroxy-3-methoxybenzylidene) thiophene-2-carbohydrazide (H_2_L). Specifically, we investigated Cu_4_L_4_’s impact on ROS generation and apoptosis induction in human OS cells using 2D and 3D MG-63-derived cell models, as well as xenografts. Additionally, we explored the effect of sustained Cu_4_L_4_ administration on OS tumor progression.

## 2. Materials and Methods

### 2.1. Materials

Dulbecco’s modified Eagle’s medium (DMEM) and TrypLE™ were purchased from Gibco (Gaithersburg, MD, USA). Fetal bovine serum (FBS) was bought from Internegocios S.A. (Mercedes, Argentina). Mouse-derived fibroblasts (L929) and OS (MG-63) cell culture lines were acquired from the American Type Culture Collection (ATCC). Tissue culture materials were purchased from Jet Bio-Filtration Co. (Guangzhou, China). Annexin V-Fluorescein isothiocyanate (FITC), propidium iodide (PI), and tetrazolium salt MTT (3-(4,5-dimethylthiazol-2-yl)-2,5-diphenyl-tetrazoliumbromide) were supplied by Invitrogen Co. (Buenos Aires, Argentina). Agarose was purchased from Inbio Highway (Tandil, Argentina). Resazurin was purchased from Santa Cruz Biotechnology (Dallas, TX, USA). Other reagents were of analytical or HPLC grade from available commercial sources and used as received from Merck (Darmstadt, Germany) or a similar brand.

### 2.2. Methods

#### 2.2.1. Synthesis and Characterization of Cu_4_L_4_ Complex

Ligand H_2_L and Cu_4_L_4_ complex were prepared following the procedure reported in our previous work, and characterized by a variety of physicochemical methods [34]. To determine the stability of the Cu_4_L_4_ solution, the electronic spectrum was measured at different times using a Shimadzu UV-2006 spectrophotometer (Shimadzu Corporation, Kyoto, Japan). UV-Vis spectra were recorded in a solution of dimethyl sulfoxide (DMSO) at 1.25 × 10^−5^ and 1.25 × 10^−3^ mol L^−1^ from 0 to 24 h using 10 mm quartz cells in the spectral range from 300 to 900 nm. Dimethyl sulfoxide (DMSO) was used to prepare H_2_L (5 mM, MW = 328.8 g mol^−1^) and Cu_4_L_4_ stock solutions (2.5 mM, MW = 1489.1 g mol^−1^, green solution), which were then forward-diluted in cell culture medium based on the concentrations required in each biological experiment. The maximum DMSO concentration was maintained at 0.5 percent.

#### 2.2.2. Cell Culture Conditions

Human OS (MG-63) and mouse-derived fibroblast (L929) cell lines were cultured in DMEM supplemented with 10% FBS, 100 IU/mL penicillin, and 100 µg/mL streptomycin at 37 °C in 5% CO_2_ atmosphere.

#### 2.2.3. Cell Viability

Cytotoxic study was performed according to Mosmann [35]. Cells were seeded in a 96-well plate at a density of 3.5 × 10^4^ cells/mL, allowed to attach for 24 h and then treated with 0.5% DMSO in DMEM (Control) or different concentrations of ligand (1–25 µM) and complex (0.15–1.5 µM) at 37 °C for 24 h. Afterward, the medium was replaced and the cells were incubated with 0.5 mg/mL MTT under normal culture conditions for 3 h. Cell viability was manifested by the conversion of the tetrazolium salt MTT to a colored formazan by mitochondrial dehydrogenases. Color development was measured spectrophotometrically with a microplate reader (Multiplate Reader Multiskan FC, Thermo Scientific, Waltham, MA, USA) at 570 nm after cell lysis in DMSO (100 µL per well). Cell viability was plotted as the percentage of the control value.

#### 2.2.4. Clonogenic Assay

To determine the effect of Cu_4_L_4_ on the reproductive potential of MG-63 cells, a clonogenic experiment was carried out, following the protocol reported by Franken et al. [36]. Cells were plated in a 12 well-dish at low density (200 cells/well) and were treated with different concentrations of complex at a range of 0.0125–0.025 µM. After 24 h, the cells were washed with phosphate-buffered saline (PBS, pH 7.4) and 1 mL of DMEM supplemented with 10% FBS was added. Half of the medium was taken out of the plates and replaced with fresh complete medium every two days for a total of eight days of incubation at 37 °C. After this time, cells were stained with a mixture of 6% of glutaraldehyde and 0.5% of crystal violet for 30 min at room temperature, washed with distilled water and dried. The plating efficiency (PE) is defined as the ratio of the number of colonies to the number of cells seeded (Equation (1)) whilst the number of colonies that survive after treatment, expressed in terms of PE, is called the surviving fraction (SF) (Equation (2)) [36].

PE = (cell colonies control/cell seeded) × 100,(1)

SF = [cell colonies/(PE × cell seeded)] × 100,(2)

#### 2.2.5. Reactive Oxygen Species (ROS) Study

Oxidative stress on MG-63 cells was evaluated by measuring the intracellular production of ROS after treating the cell monolayer with different concentrations of Cu_4_L_4_. Briefly, the cells were grown in 24-well plates (10^5^ cells/well), incubated for 30 min in the dark with dihydrorhodamine-123 (DHR-123), and then treated for 3 h with Cu_4_L_4_ at 37 °C in darkness. ROS generation was determined by the oxidation of DHR-123 to rhodamine by spectrofluorescence at 530 nm. Protein content was measured using the Pierce^TM^ BCA Protein Assay Kit (Thermo Fisher Scientific, Waltham, MA, USA), and the results were adjusted accordingly.

#### 2.2.6. Apoptosis Assay

Cells in early and late stages of apoptosis were detected with Annexin V-FITC and PI staining [37]. Cells were treated with DMEM alone (Control) or Cu_4_L_4_ (0.4 to 0.6 µM) for 24 h prior to analysis. For the staining, cells were washed with PBS and staining with Annexin V/PI (100 µL of cell suspension on binding buffer, 1 µL of Annexin V-FITC and 1 µL PI 2 mg/mL). Cells were analyzed using a flow cytometer BD Accuri C6 Plus and BD Accuri C6 Plus software version 1.0.23.1. For each analysis, 10,000 counts, gated on an FSC vs. SSC dot plot, were recorded.

#### 2.2.7. 3D Studies

##### 2.2.7.1. Multicellular Tumor Spheroid Formation

MG-63 cells were used to create multicellular tumor spheroids using the modified hanging drop technique that we had previously presented [38]. Briefly, on the cell culture plate lid, 25 µL drops of cell suspension containing 400 cells each were suspended. Following the 72 h period needed for cell aggregation, the spheroids were transferred to 96-well plates coated with agarose (one droplet per well) and cultivated using 150 µL of complete media. Spheroids were allowed to grow for another 48 h until reaching a size of 400 µm diameter.

##### 2.2.7.2. Spheroid Cell Viability Studies

Multicellular spheroids (MCS) were treated in 96-well plates with 0.5% DMSO in DMEM (Control) and with Cu_4_L_4_ in a range of concentration from 1.5 to 9.5 µM in DMEM for 24 h. Afterward, cell viability was evaluated by the resazurin reduction assay, in which resazurin dye is irreversibly reduced by intracellular oxidoreductases to a pink-red fluorescent dye known as resorufin [39]. Fluorescence was registered using a fluorometer Shimadzu RF-6000 (Shimadzu Corporation, Kyoto, Japan) (excitation at 560 nm, emission at 590 nm). Cell viability was plotted as a percentage of the basal condition (solvent control).

Furthermore, morphological changes were examined using a live–death cell labeling method that we have previously described [40]. After being incubated for 24 h with 0.5% DMSO in DMEM (Control) or varying doses of Cu_4_L_4_ (2.5–7.5 µM), MCS were stained with fluorescein diacetate (FDA, 8 × 10^−3^ mg/mL) and PI (2 × 10^−2^ mg/mL), and incubated in the dark for 5 min at room temperature. The Z-stack fluorescence images of the spheroids was conducted on a Carl Zeiss Observer LSM 800 Confocal Microscope with a 10X EC plan-neofluar M27 (NA 0.3) air objective operated by ZEN 2.1 software, using 561 nm and 488 nm laser wavelength with 188 µm and 31 µm pinhole, respectively. Fiji-ImageJ software version 2.14.0 was used to process the raw photos, producing composite RGB images.

##### 2.2.7.3. MCS Spreading Assay

To assess the capacity of the spheroids’ cells to migrate and proliferate following a 24 h exposure to Cu_4_L_4_, the spheroids were transferred into a 48-well plate containing 500 µL of 0.5% DMSO in DMEM (Control) or with different concentrations of complex (1.5–6.5 µM) and incubated under normal conditions. Following the attachment of the spheroid to the plastic surface, the cells began to migrate, expanding the attachment region concentrically in the process. Following a 24 h treatment, the cells were fixed with methanol, stained with Giemsa (20%), and an inverted microscope was used to measure the cell migration.

#### 2.2.8. In Vivo

##### 2.2.8.1. In Vivo OS Tumor Growth

Four-weeks-old outbred male N:NIH(S)-nu mice were acquired from the Animal Facility of the School of Veterinary Sciences at the Universidad Nacional de La Plata (Buenos Aires) and housed in the Universidad Nacional de La Plata animal facility, with free access to water and food. To create tumor implants, a cell suspension containing 5 × 10^6^ MG-63 cells and 100 µL of DMEM was injected subcutaneously (s.c.) into the right flank of 6-weeks-old nude mice. Animals were randomly assigned to a control group (PBS) and a Cu_4_L_4_ therapy group following tumor cell injection, 7 animals per group.

Three days after the tumor challenge, when all tumors were palpably confirmed, treatment with Cu_4_L_4_ (2 mg/kg intraperitoneal, i.p.) was initiated and administered twice weekly for 5 weeks. The weight of the animals and the growth of the xenograft were recorded throughout the experiment twice a week, from day 3 (before treatment) until necropsy on day 45. A caliber was used to measure the xenograft growth, and the following Equation (3) was used to calculate the volume:

V = 0.52 × W^2^ × L, (3)

where W = width and L = length. The linear regression slopes of the xenograft volumes over time (days 3–45) were used to calculate the tumor growth rates (TGR) for each experimental condition.

When primary tumors reached the maximum volume limit of 800 mm^3^ and started showing ulceration and signs of skin invasion, animals were euthanized by CO_2_ inhalation, and then blood samples were obtained by cardiac puncture for further analysis. Mice were photographed after sacrifice and protocol termination.

##### 2.2.8.2. Histopathological Studies in OS Xenografts

Histopathological assessment of OS tumors involved mitotic index quantification in viable sections of hematoxylin and eosin (H&E)-stained tumor slides and determination of adjusted tumor necrotic rate after treatment. Mitotic bodies in H&E-stained slides were counted in randomly selected high-power field (HPF) at X400 magnification. Only viable sections of tumor tissue were analyzed for mitotic index calculation. Histological analysis was performed and confirmed by two blinded researchers. For tumor necrosis assessment, color brightfield images of entire H&E-stained tumor sections were acquired at X2.5 magnification using a Cytation Gen5 Reader (BioTek, Winooski, VT, USA). Images were collected using a 4 × 5 grid and the stitching was performed with the “Image Montage” function setting a tile overlapping of 10%. Necrotic area in tumor tissue sections was measured using ImageJ 1.5j8 Software (NIH, Bethesda, MD, USA). Tumor necrosis was identified as tissue areas with a marked increase in eosinophilia and quantification of both necrotic area (NA) and viable area (VA) was performed using the “Color Threshold” tool. Tumor necrotic rate (TNR) was then calculated with Equation (4) in 4 sections per experimental group.

TNR = (NA × 100)/(VA + NA),(4)

Adjustment of % of necrotic areas to changes in tumor size and determination of adjusted tumor necrotic rate (ATNR) was performed using Equation (5):

ATNR = 100 − (100 − TNR) × RTGR,(5)

where RTGR stands for group-specific relative tumor growth rates. RTGR was obtained after transforming TGR values (8.6 ± 0.8 and 6.0 ± 1.0 mm^3^/day for Control and Cu_4_L_4_, respectively), taking the TGR of the control group as “1”.

##### 2.2.8.3. Toxicological Studies

Whole blood samples were divided in tubes coated with heparin and tubes without anticoagulant reagent after concluding the in vivo procedure and killing the animals, in order to conduct additional hematological and biochemical analysis. Aspartate aminotransferase (GOT) and alanine aminotransferase (ALT) activity were measured, together with the levels of creatinine and total protein. Red and white blood cell counts, hematocrit, and platelet counts were also conducted. Furthermore, following euthanasia, liver, heart, lung, kidneys and spleen were recovered, fixed and processed for histopathological assessment, after H&E staining.

##### 2.2.8.4. Ethics Statements

This protocol was approved by the Institutional Animal Care and Use Committee of the School of Exact Sciences at the Universidad Nacional de La Plata (Protocol 003-00-25).

Findings from in vivo protocols were reported according to the ARRIVE guidelines. Assays were carried out in accordance with the Guide for the Care and Use of Laboratory Animals (NIH Publications No. 8023; Rev. 1978).

#### 2.2.9. Statistical Analysis

Three independent experiments were achieved and the results are expressed as mean ± standard error of the mean (SEM), unless stated otherwise. The analysis of variance method (ANOVA) was used to examine statistical differences, and the test of least significant difference (Fisher) was used after that. *t* tests were used for non-parametric and normal distribution of data. Differences were considered statistically significant at a level of *p* < 0.05. The statistical analyses were performed using GraphPad Prism v8.0.1 (GraphPad Software Inc., www.graphpad.com).

## 3. Results

### 3.1. Synthesis and Characterization of Cu_4_L_4_ Complex

**Figure 2:**
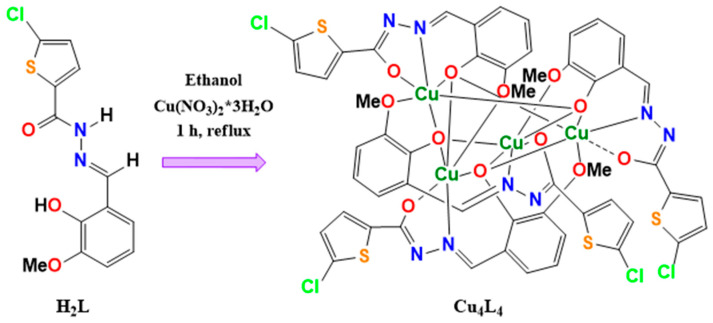
Schematic procedure of synthesis of Cu_4_L_4_ complex from H_2_L ligand.

The Cu_4_L_4_ complex was synthetized from H_2_L hydrazone (see Figure 2) and fully characterized in our previous work [34]. The UV-vis electronic spectrum of the complex was monitored at different time intervals to assess its stability in solution and compared with the solid-state spectrum. As shown in Figure S1, there are no differences between the solid and solution spectra, so it can be concluded that the coordination environment of Cu in solution did not change significantly over a 24-h period. The slight differences in the absorption maxima between the solid and solution spectra (see Table S1) are attributed to the different methods used for their acquisition, suggesting that the coordination environment remains essentially unchanged from the solid state to solution.

### 3.2. Cell Viability

Cytotoxicity studies for H_2_L and Cu_4_L_4_ on human OS MG-63 cell line were performed using MTT assay. As can be seen in Figure S2, Cu_4_L_4_ demonstrated potent anticancer activity on the OS cells from 0.3 µM (*p* < 0.0001), in a dose-dependent manner showing an IC_50Cu4L4_ of 0.50 ± 0.04 µM after 24 h of incubation. Although H_2_L was also proven to reduce cell viability, its effect was 35-fold lower than that of the complex (IC_50H2L_ = 17.8 ± 2.4 µM), highlighting the significance of complexation with metal atoms to modulate the anticancer activity of the bioactive ligands, as we previously reported [28,41,42]. Moreover, Cu_4_L_4_ and H_2_L exhibited stronger antitumor effects than CDDP (IC_50CDDP_ = 39 ± 1.8 µM). We also evaluated the effect of H_2_L and Cu_4_L_4_ on L929 cells (mouse-derived fibroblasts) and compare the selectivity index (SI = IC_50_ non-tumoral cells/IC_50_ tumor cells) of the complex with that of the CDDP. The results showed that the complex has a greater selectivity for the tumor cells (SI = 2.1) than the CDDP (SI = 0.3). Similar results were observed for H_2_L and Cu_4_L_4_ after 48 and 72 h of incubation with IC_50_ around 0.4 and 4 for complex and ligand, respectively (Table S1).

Numerous scientific studies indicate that copper complexes with IC_50_ values in the low micromolar range (<10 µM) are efficient cytotoxic agents against a variety of human cancer cell types [43,44]. As mentioned above, our group reported a series of Cu(II)-hydrazone complexes with potent cytotoxic activity against MG-63 cells (Table 1). These complexes are structurally related to Cu_4_L_4_ via the aldehyde moiety of the ligand, 2-hydroxy-3-methoxybenzaldehyde (o-HVA), as shown in Figure 1. However, the structural differences in the hydrazide group of the ligand are reflected in the potency of their cytotoxic activity in this cell line (furan > thiophen = methoxi-benzohydrazide > hydroxy-benzohydrazide) [27,28,31,32]. Additionally, we found that while co-ligands like o-phen and bipy are capable of increasing the complex’s activity in this cell line, as in the case of CuHL4, they still do not match the potency that the hydrazide exchange produces [22]. Notably, the addition of the halogen Cl in the case of Cu_4_L_4_ was shown to increase the potency of the cytotoxic effect by four times compared to CuHL1, surpassing the effect of the change from thiophene to furan in CuHL2. This enhanced activity observed for chlorinated complex (Cu_4_L_4_) is due to increased lipophilicity that facilitates cell membrane and spheroid permeation. In addition, the potential modulation of ligand field strength influences intracellular copper release dynamics.

**Figure 3:**
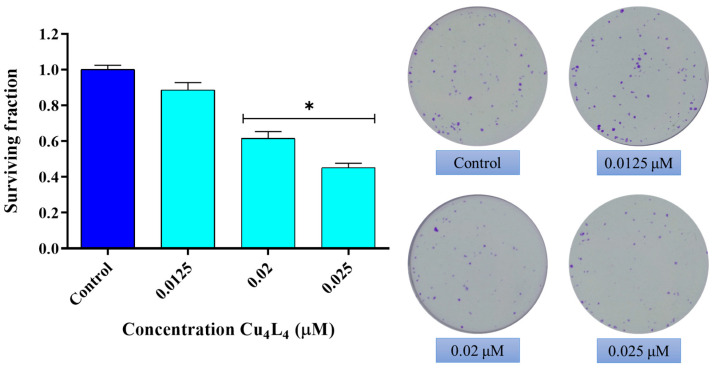
Effect of Cu_4_L_4_ on MG-63 cell reproductive potential. Cells were incubated in DMEM alone (Control) or with several concentrations (0.0125, 0.02 and 0.025 µM) of complex for 24 h. The results are expressed as surviving fraction (percentage of the basal level) and represent the mean ± SEM (n = 12). * *p* < 0.0001 differences between Control and treatment.

We further evaluate the effect of Cu_4_L_4_ on the cellular reproductive potential using a clonogenic study. As can be seen in Figure 3, a reduction in cell proliferation of MG-63 cells occurs after 24 h treatment with Cu_4_L_4_ from 0.02 µM with a surviving fraction (SF) of 0.6 to an SF of 0.45 at 0.025 µM (*p* < 0.0001). It should be highlighted that the concentrations utilized are significantly lower than its IC_50_ presented above.

### 3.3. Oxidative Stress

The relationship between redox potential and the anticancer activity of copper compounds has been established in several scientific studies due to the accumulation of ROS caused by Fenton reactions initiated by copper complexes [46,47]. Therefore, we used the DHR-123 probe, which selectively reacts with hydrogen peroxide and peroxynitrite, to examine the complex’s potential impact on ROS formation [48,49]. Following cellular uptake, intracellular ROS oxidize DHR-123 to fluorescent rhodamine 123. The 3 h time point was selected to capture early redox imbalance events prior to the onset of secondary apoptotic processes. This approach allows discrimination between primary ROS generation and downstream oxidative damage.

**Figure 4:**
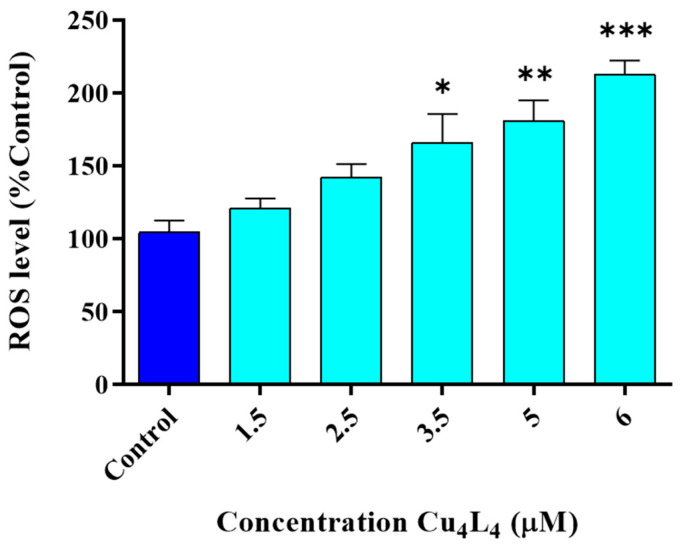
Generation of intracellular ROS in MG-63 cells after 3 h treatment with Cu_4_L_4_. The results of fluorescence were corrected by their protein content and are expressed as the % of basal level (Control) as the mean ± SEM (n = 12). * *p* < 0.01, ** *p* < 0.001 and *** *p* < 0.0001 differences between Control and treatment.

We demonstrated that Cu_4_L_4_ causes a significant dose-dependent increase in intracellular ROS levels in comparison to Control from 3.5 µM (160%) to 6 µM (215%) after a 3 h treatment (Figure 4). This result suggests that the generation of oxidative stress, at least partly, could be one of the important mechanisms of action of the complex. However, we also reported this behavior for other Cu(II)-hydrazone complexes in MG-63 cells, indicating that this family of compounds shares the same mechanisms of action [28,31].

### 3.4. Apoptosis

Apoptosis is a type of programmed cell death that causes damaged cells to be removed in an orderly and effective way. It can be initiated by extrinsic signals, such as ligand binding to cell surface death receptors or in the presence of harmful agents, or by internal signals like genotoxic stress. A hallmark of cancer that contributes to both tumor formation and progression as well as tumor resistance to treatment is deregulation of the apoptotic cell death mechanism. In clinical oncology, the majority of anticancer medications currently in use employ intact apoptotic signaling pathways to cause cancer cell death [50].

Regardless of the kind of cell or harmful agent, the externalization of phosphatidylserine is an essential marker of prior apoptotic processes. In this context and with the aim of investigating whether Cu_4_L_4_ induces apoptosis in MG-63 cells, we performed a flow cytometry assay using Annexin V, which specifically binds to phosphatidylserine, and propidium iodide (PI) [37]. Table 2 and Figure S3 display the flow cytometry results of the apoptotic process in the presence of complex Cu_4_L_4_ from 0.4 to 0.6 µM on MG-63 cells. As we previously observed for other comparable complexes [28,31,32], we found a significantly increase in necrotic and late apoptotic cell populations at all tested doses of Cu_4_L_4_.

### 3.5. 3D Studies

#### 3.5.1. Spheroid Cell Viability Studies

Although 2D cultures are the first step in assessing a compound’s cytotoxicity, 3D cultures offer a more accurate representation of the intratumoral environment, including gradients of nutrients and oxygen, as well as cell–cell interactions [51]. Multicellular resistance (MCR) in solid tumors can also be replicated using 3D models, such as MCS, which affect the sensitivity of cells to therapies and occur as a result of cell–cell interaction, limited drug penetration, and the resistance of quiescent cells found in the MCS’ core [52]. Due to its impact on apoptotic factors expression, hypoxia has also been implicated as a cause of MCR [53].

Therefore, to continue studying the anticancer activity of the complex Cu_4_L_4_, we generated MCS from MG-63 cells by the hanging drop technique [38]. Our results indicated that Cu_4_L_4_ presented an IC_50_ of 4.11 ± 0.12 µM, which corresponds to a cytotoxic effect 15-fold greater than CDDP (IC_50_ = 65.1 ± 5.6 µM). In addition, the complex had a dose-dependent effect on the spheroids’ volume and shape (see Figure S4). This potent anticancer effect in 2D and 3D cultures surpassed the effects produced by other Cu(II) hydrazone compounds previously reported by our group [28,31,32], indicating that this family of compounds has promising potential for cancer therapy (see Table 3).

**Figure 5:**
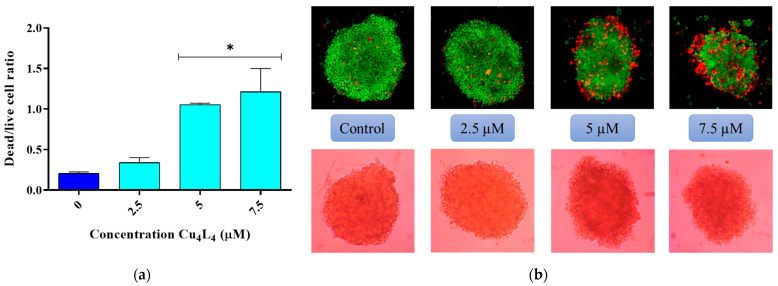
(**a**) Graphical representation of the ratio of dead/live cells in OS spheroids treated with 0.5% DMSO in DMEM (Control) or different concentrations of Cu_4_L_4_ (2.5–7.5 µM) for 24 h. Results are expressed as the mean of the dead/live cell ratio ± SEM from three independent experiments. * *p* < 0.01 differences between control and treatment. (**b**) Representative images of the MCS treated with Cu_4_L_4_, before (bottom panel) and after (upper panel) the live–dead staining with fluorescein FDA and PI.

Moreover, we studied the morphologic changes in the MCS after Cu_4_L_4_ treatment with a live/dead staining with FDA and PI. As shown in Figure 5, the ratio of dead/live cells increased with increasing concentrations, as the loss of MCS integrity was observed, which correlates with the results obtained for cell viability.

#### 3.5.2. Spheroid Cell Viability Studies

**Figure 6:**
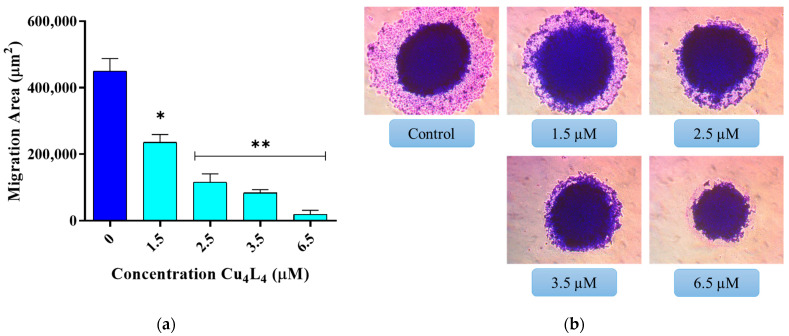
Spreading assay of spheroids. MCS were incubated 24 h with 0.5% DMSO in DMEM (Control) or different concentrations (1.5−6.5 µM) of Cu_4_L_4_. (**a**) The results are expressed as the migration area and represent the mean ± SEM. * *p* < 0.001 and ** *p* < 0.0001 differences between control and treatment. (**b**) Representative images of the spheroids after fixed and staining process.

The most common cause of cancer-related mortality is metastasis, which is the dissemination of cancer cells from the original tumor to surrounding tissues and to other organs [54]. Numerous factors contribute to metastasis, including cell migration via blood or lymph arteries, infiltrative growth through the extracellular matrix (ECM), and the emergence of distant colonies [55]. A spreading experiment can be used to closely simulate the migration of cells out of small cancer clusters in terms of tumor biology. To investigate the effect of Cu_4_L_4_ on the migration of spheroid cells, MCS derived from MG-63 cells were treated with concentrations ranging from 1.5 to 6.5 µM of complex. We found that the average migration area of the basal condition is 450,000 µm^2^, whereas the Cu_4_L_4_ treatment causes a decrease on this value from 235,000 µm^2^ at 1.5 µM to 19,000 µm^2^ at 6.5 µM (see Figure 6). Importantly, the complex’s impact starts at sub-IC_50_ concentrations, a phenomenon we reported previously for another Cu(II)-hydrazone complex on MG-63 cells-derived MCS [28]. These findings support the compound’s inhibitory effects on OS spheroid’s cell migration.

### 3.6. In Vivo Study

**Figure 7:**
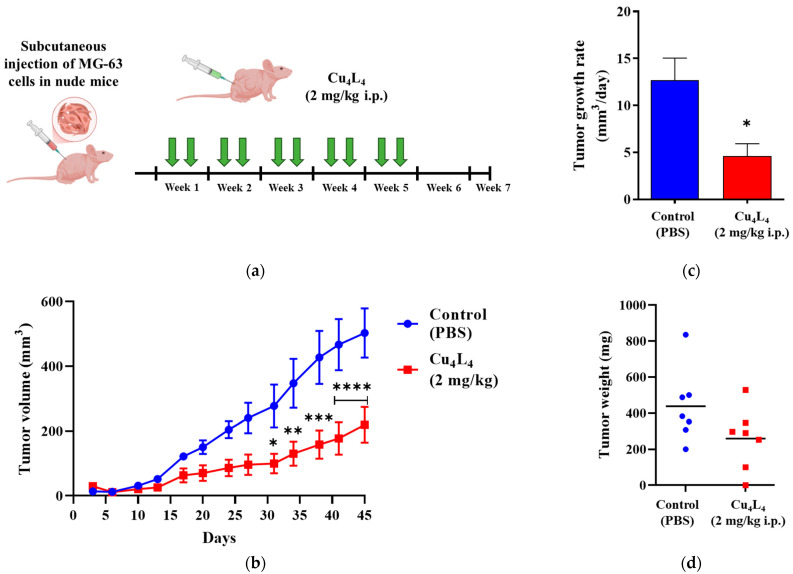
(**a**) Experimental design of the in vivo protocol carried out to assess the activity of Cu_4_L_4_, administered using a 2 mg/kg i.p. dose, twice a week (green arrows), on human OS MG-63 xenograft progression in N:NIH(S) nude mice. (**b**) Curves represent mean tumor volumes of mice receiving Control (blue) or Cu_4_L_4_ (red) treatments, seven male mice per group. (**c**) Tumor growth rates calculated for Control (blue) and Cu_4_L_4_ (red) treatments from tumor volumes (between days 3 and 45). (**d**) Tumor burden additionally assessed by weighing OS primary lesions after necropsy and tumor recovery. The results are expressed as the mean ± SEM. * *p* < 0.05, ** *p* < 0.01, *** *p* < 0.001 and **** *p* < 0.0001 differences between Control and treatment.

The positive outcomes of the 2D and 3D tests led us to investigate whether the anticancer impact of Cu_4_L_4_ was replicated in vivo. The complex activity was evaluated on MG-63 xenografts growing in nude mice, at a dose of 2 mg/kg (0.3 mM) administered i.p., two times/week, after tumor engraftment confirmation (Figure 7a). As shown in Figure 7b, Cu_4_L_4_ treatment caused a reduction in tumor volume from 502.6 ± 76.3 mm^3^ to 219.5 ± 55.0 mm^3^ (mean ± SEM), which corresponds to a 43.6% inhibition of tumor growth compared to animals treated with PBS (Control group). Furthermore, this effect can also be observed when evaluating the tumor growth rate, which is two times lower in the group treated with the complex compared to that obtained for the Control group (see Figure 7c). Despite these findings, the decrease in tumor weight in Cu_4_L_4_ treated mice was not statistically significant due to the high dispersion of the samples (Figure 7d). Comparable outcomes were obtained for CuHL1 when tested in nude mice bearing OS xenografts with a 4-week treatment schedule (three times per week) [32].

**Figure 8:**
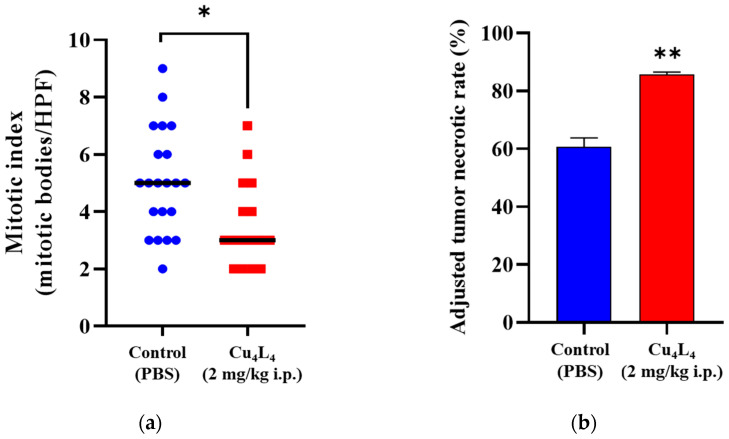
(**a**) OS tumor slices from Control or Cu_4_L_4_-treated mice were evaluated for mitotic index. Information was presented as the number of HPF/mitotic bodies. (**b**) Following PBS (Control) or Cu_4_L_4_-treatment, necrosis in primary OS lesions was also assessed and displayed as ATNR. The results are expressed as the mean ± SEM. * *p* < 0.05, ** *p* < 0.0001 differences between Control and Cu_4_L_4_-treatment.

Figure S5 displays representative images of mice harboring OS xenografts from the Control (PBS) or Cu_4_L_4_ treated groups. Mitotic indexes were measured in MG-63 lesions, given their prognostic significance in cancer patients and their correlation with OS aggressiveness. The results showed that the Control and Cu_4_L_4_ MG-63 lesions had mitotic bodies/HPF values of 5.0 ± 0.4 and 3.6 ± 0.4 (mean ± SEM), respectively (see Figure 8a and Figure S6). Moreover, necrosis in OS xenografts was evaluated using the ATNR method. As shown in Figure 8b and Figure S7, Cu_4_L_4_ treatment cause an increase in the ATNR (85.7 ± 0.9) compared to the basal level of the PBS-treated group (60.7 ± 3.0). These findings are in accordance with the reduction in the tumor volume and the tumor growth rate obtained for the Cu_4_L_4_ treated group. In addition, comparable mitotic index and necrosis rate values were previously documented for CuHL1 by our group [32]. Notably, a mouse in the Cu_4_L_4_-treated group showed a reduction in tumor size from week three of treatment until it completely regressed in week four, staying in remission until the end of the trial.

**Figure 9:**
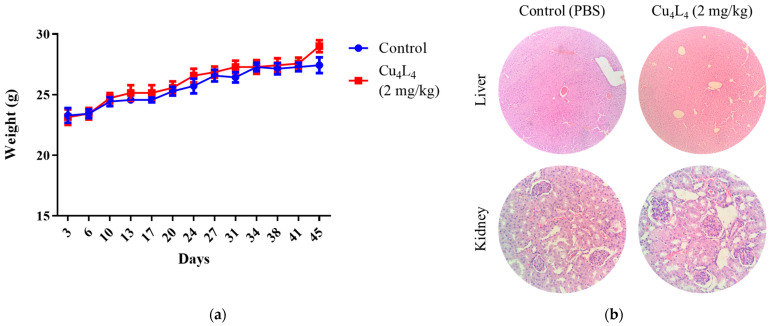
(**a**) The toxicity of Cu_4_L_4_ treatment was verified by tracking the animal weight of Control or Cu_4_L_4_-treated nude mice carrying OS xenografts during the in vivo test. (**b**) Representative images of H&E-stained liver and kidney slides belonging to animals treated with PBS (Control) or Cu_4_L_4_. Images of liver and kidney were taken at ×100 and ×400 magnification, respectively.

The general health of the animals was also evaluated throughout the trial. During the experiment, no alterations in behavior or food and water consumption were noted. All animals’ weights increased during the trial as a result of their growth, and there were no significant differences between the Control and complex-treated groups (see Figure 9a). In renal tissue, extensive areas of acute tubular necrosis were found on the Cu_4_L_4_-treated group (see Figure 9b). The liver, spleen and heart samples showed no significant histological changes or indications of toxicity (see Figure 9b and Figure S8). Lungs of Cu_4_L_4_-treated mice presented generalized vascular congestion, with areas of peribronchial intraalveolar hemorrhage (see Figure S8). It is well known that chemotherapy can promote pulmonary hemorrhage in the context of capillary injury and diffuse alveolar damage, but these effects can be reversible upon discontinuation of the drug and sometimes require treatment with corticosteroids [56,57].

Finally, Table 4 and Table 5 display the biochemical and hematological outcomes that were measured at the conclusion of the experiment. No changes were detected in creatinine (kidney function indicator) and total protein values, hematocrit values, or absolute leukocyte counts. However, a decrease in liver enzyme levels alanine aminotransferase (ALT) and aspartate aminotransferase (GOT) were observed in the Cu_4_L_4_-treated group. Despite this non-significant difference, the H&E staining results suggest no hepatic damage (see Figure 9b), and the animals evaluated remained healthy throughout the experimental period. These metrics reveal that the animals responded effectively to treatment with the compound and did not exhibit any relevant toxicity.

## 4. Conclusions

This study comprehensively evaluated the in vitro and in vivo antitumor potential of the copper(II)-hydrazone complex, Cu_4_L_4_, against human osteosarcoma. Our results conclusively demonstrate that the Cu_4_L_4_ complex is a highly effective and promising anticancer agent, surpassing the activity of its uncoordinated ligand and the standard chemotherapy drug, CDDP.

The complex demonstrated potent cytotoxicity in MG-63 OS cells, with a significantly lower IC_50_ than CDDP (0.50 ± 0.04 vs. 39 ± 1.8 µM). In addition, the complex anticancer effect was 27-fold higher than that of the ligand, highlighting the significance of complexation with metal atoms to modulate the anticancer activity of the bioactive ligands. The compound also exhibited greater selectivity for tumor cells compared to non-tumor cells (SI = 2.1 vs. SI = 0.3). The mechanism of action of Cu_4_L_4_ appears to be primarily mediated by the induction of ROS, which triggers apoptosis.

Furthermore, MCS models, which better replicate the complexity of solid tumors, showed a remarkably greater sensitivity to Cu_4_L_4_ than to CDDP (IC_50_ of 4.11 ± 0.12 vs. IC_50_ = 65.1 ± 5.6 µM). Importantly, at sub-IC_50_ concentrations, the complex significantly inhibited spheroid cell migration (control 450,000 µm^2^ vs. Cu_4_L_4_ treatment 235,000–19,000 µm^2^), suggesting a potential to prevent metastasis, a key factor in the high mortality of osteosarcoma.

The in vitro findings were successfully translated to an in vivo model, where treatment with Cu_4_L_4_ in mice with OS xenografts resulted in a 43.6% reduction in tumor volume and halving of its growth rate. These antitumor effects correlated with a significant reduction in mitotic index (mitotic bodies/HPF values 5.0 ± 0.4 and 3.6 ± 0.4) and a substantial increase in tumor necrosis with ATNR (85.7 ± 0.9) compared to the basal level of the PBS-treated group (60.7 ± 3.0). Notably, one mouse in the treated group experienced complete tumor remission.

While some signs of toxicity were observed, such as acute tubular necrosis in the kidneys and vascular congestion in the lungs, the animals showed no weight changes or systemic hepatic or hematological toxicity, which underscores the viability of the complex as a therapeutic agent.

In conclusion, this study presents the Cu_4_L_4_ complex as a promising candidate for future osteosarcoma therapies. Its potent cytotoxic activity, induction of apoptosis, ability to inhibit metastasis, and in vivo efficacy position it as a superior alternative to platinum-based treatments.

## Figures and Tables

**Table 1:** IC_50_ (µM) values Cu(II)-complexes and their ligands on MG-63 cells at 24 h of incubation.

Compound	2D	Reference
H_2_L	17.8 ± 2.4	This work
Cu_4_L_4_	0.50 ± 0.04	This work
H_2_L1	>100	[32]
CuHL1	2.1 ± 0.3	[32]
H_2_L2	>100	[28]
CuHL2	1.1 ± 0.1	[28]
H_2_L3	>100	[31]
CuHL3	2.6 ± 0.3	[31]
H_2_L4	>100	[27]
CuHL4	8.8 ± 0.3	[27]
CuL4(o-phen)	3.5 ± 0.3	[22]
CuHL4(bipy)	5.6 ± 1.0	[22]
CDDP	39.0 ± 1.8	[45]

**Table 2:** Percentage of apoptosis and necrosis populations treated with Cu_4_L_4_ (0.4–0.6 µM) for 24 h. The results are expressed as the mean ± SEM, obtained from three independent experiments. * *p* < 0.05, ** *p* < 0.001, *** *p* < 0.0001 differences between Control and treatment.

Concentration	AnnV−/PI−	AnnV+/PI−	AnnV−/PI+	AnnV+/PI+
0 µM	90.2 ± 0.7	6.2 ± 1.2	1.7 ± 0.5	2.6 ± 0.3
0.4 µM	52.5 ± 5.6 ***	8.2 ± 1.2	24.9 ± 10.7 **	20.9 ± 2.1 *
0.5 µM	50.9 ± 6.5 ***	6.1 ± 0.8	26.4 ± 8.7 **	19.2 ± 2.6 *
0.6 µM	43.9 ± 6.6 ***	10.4 ± 1.1	18.4 ± 5.3 *	27.3 ± 1.2 **

**Table 3:** IC_50_ (µM) values of Cu_4_L_4_ and our Cu(II)-hydrazones complexes previously reported on monolayers and MCS derived from MG-63 cells at 24 h of treatment. n.d: not determinate.

Cu(II)-Complexes	2D	3D	Reference
Cu_4_L_4_	0.50 ± 0.04	4.11 ± 0.12	This work
CuHL1	2.1 ± 0.3	9.1 ± 1.0	[32]
CuHL2	1.1 ± 0.1	16.3 ± 3.1	[28]
CuHL3	2.6 ± 0.3	9.9 ± 1.4	[31]
CuHL4	8.8 ± 0.3	n.d	[27]
CDDP	39.0 ± 1.8	65.1 ± 5.6	[45]

**Table 4:** Biochemical and general hematological parameters in mice after sustained treatment with Cu_4_L_4_ in comparison to Control (PBS-treated) animals.

Treatment	Weight (g)	Hematocrit (%)	RBC (10^6^/µL)	WBC (10^3^/µL)	Total Protein (g/dL)	Creatinine (mg/mL)	GOT (IU/L)	ALT (IU/L)
Control	27.4 ± 0.7	41.5 ± 1.6	7.5 ± 1.7	4.3 ± 1.1	5.4 ± 0.1	0.8 ± 0.4	211.8 ± 84.9	129.7 ± 64.1
Cu_4_L_4_	29.0 ± 0.5	44.3 ± 0.6	8.0 ± 0.1	4.5 ± 0.5	5.4 ± 0.2	0.6 ± 0.1	99.1 ± 19.8	63.6 ± 22.5

GOT: aspartate aminotransferase, ALT: alanine aminotransferase.

**Table 5:** Absolute leukocyte counts in mice after sustained treatment with Cu_4_L_4_ in comparison to PBS-treated (Control) animals.

Treatment	Band Neutrophils	Segmented Neutrophils	Eosinophils	Basophils	Lymphocytes	Monocytes
Control	1.8 ± 1.3	59.5 ± 13.3	1.5 ± 1.1	0	33.0 ± 8.6	4.3 ± 2.5
Cu_4_L_4_	1.6 ± 0.5	52.0 ± 4.8	2.3 ± 0.6	0	40.4 ± 4.3	3.6 ± 1.5

## Data Availability

The original contributions presented in this study are included in the article/Supplementary Materials. Further inquiries can be directed to the corresponding author.

## Supplementary Materials

The following supporting information can be downloaded at: https://www.mdpi.com/article/10.3390/pharmaceutics18030372/s1, Figure S1: Electronic absorption spectra in UV−vis region of Cu_4_L_4_ in solid state and in DMSO solution from 0 to 24 h; Figure S2: Cytotoxic effect of H_2_L and Cu_4_L_4_ on MG-63 cells was evaluated by MTT assay; Figure S3: Impact of Cu_4_L_4_ on MG-63 cell apoptosis induction; Figure S4: Cell viability of the MG-63 spheroids evaluated with resazurin probe; Figure S5: Representative photos of nude mice with OSA xenografts from the groups treated with Cu_4_L_4_ or Control (PBS) at day 45; Figure S6: Representative images of H&E-stained tumor slides belonging to animals treated with Control (PBS) or Cu_4_L_4_; Figure S7: Representative digitally stitched images of complete tumor sections from Control and Cu_4_L_4_ treatment groups; Figure S8: Representative images of H&E-stained spleen, heart and lung slides belonging to animals treated with PBS (Control) or Cu_4_L_4_; Table S1: Experimental electronic spectra of the complex in DMSO solution and in the solid state; Table S2: IC_50_ (µM) values of Cu_4_L_4_ and H_2_L over MG-63 cells at 48 and 72 h of treatment.

## Abbreviations

The following abbreviations are used in this manuscript:

OS	Osteosarcoma
CDDP	Cisplatin
ROS	Reactive oxygen species
DNA	Deoxyribonucleic acid
DMEM	Dulbecco’s modified Eagle’s medium
FBS	Fetal bovine serum
FITC	Fluorescein isothiocyanate
PI	Propidium iodide
MTT	3-(4,5-dimethylthiazol-2-yl)-2,5-diphenyl-tetrazoliumbromide
DMSO	Dimethyl sulfoxide
PBS	Phosphate-buffered saline
PE	Plating efficiency
SF	Surviving fraction
DHR-123	Dihydrorhodamine-123
MCS	Multicellular spheroids
FDA	Fluorescein diacetate
s.c.	Injected subcutaneously
i.p.	Intraperitoneal
TGR	Tumor growth rates
H&E	Hematoxylin and eosin
HPF	High-power field
NA	Necrotic area
VA	Viable area
TNR	Tumor necrotic rate
ATNR	Adjusted tumor necrotic rate
RTGR	Relative tumor growth rates
GOT	Aspartate aminotransferase
ALT	Alanine aminotransferase
SEM	Standard error of the mean
SI	Selectivity index
o-HVA	2-hydroxy-3-methoxybenzaldehyde
MCR	Multicellular resistance
ECM	Extracellular matrix
