# Supplementary material for: In Vitro and In Vivo Effects of a Copper(II)-Hydrazone Complex Against Human Osteosarcoma
Source: Pharmaceutics. 2026 Mar 17;18(3):372. doi: 10.3390/pharmaceutics18030372 (PMC13029629; doi:10.3390/pharmaceutics18030372)
Supplement: Supplementary file 1 [file pharmaceutics-18-00372-s001.zip › pharmaceutics-4076270-supplementary.pdf]

Article

In vitro and in vivo effects of a copper(II)-hydrazone complex against human osteosarcoma

Lucía Santa Maria de la Parra [1], Matías H. Assandri [2], Luisina M. Solernó [3,4], María de los A. Serradell [2,5], Daniel F. Alonso [3], Juan Garona [3,4], Lucía M. Balsa [1] and Ignacio E. León [1,6,*]

[1] CEQUINOR (UNLP, CCT-CONICET La Plata, asociado a CIC), Departamento de Química, Facultad de Ciencias Exactas, Universidad Nacional de La Plata (UNLP), La Plata, Buenos Aires, Argentina; luciasanta-maria@quimica.unlp.edu.ar ; luciabalsa@quimica.unlp.edu.ar ; ileon@biol.unlp.edu.ar
[2] Cátedra de Microbiología, Departamento de Ciencias Biológicas, Facultad de Ciencias Exactas, Universidad Nacional de La Plata (UNLP), La Plata, Buenos Aires, Argentina; massandri@exactas.unlp.edu.ar ; ma-serr@biol.unlp.edu.ar
[3] Centro de Oncología Molecular y Traslacional (COMTra), Universidad Nacional de Quilmes, Buenos Aires, Argentina; lusolerno@gmail.com ; danielfalonso@gmail.com ; garonajuan@gmail.com ;
[4] Unidad de Investigación Biomédica en Cáncer (IBioCAN), Centro de Medicina Traslacional, Hospital de Alta Complejidad en Red El Cruce "Dr. Néstor Carlos Kirchner" S.A.M.I.C, Florencio Varela, Buenos Aires, Argentina; lusolerno@gmail.com; garonajuan@gmail.com
[5] Instituto de Ciencias de la Salud, Universidad Nacional Arturo Jauretche (UNAJ), Florencio Varela, Buenos Aires, Argentina; maserr@biol.unlp.edu.ar
[6] Cátedra de Fisiopatología, Departamento de Ciencias Biológicas, Facultad de Ciencias Exactas, Universidad Nacional de La Plata (UNLP), La Plata, Buenos Aires, Argentina; ileon@biol.unlp.edu.ar
* Correspondence: ileon@biol.unlp.edu.ar

Supplementary Material

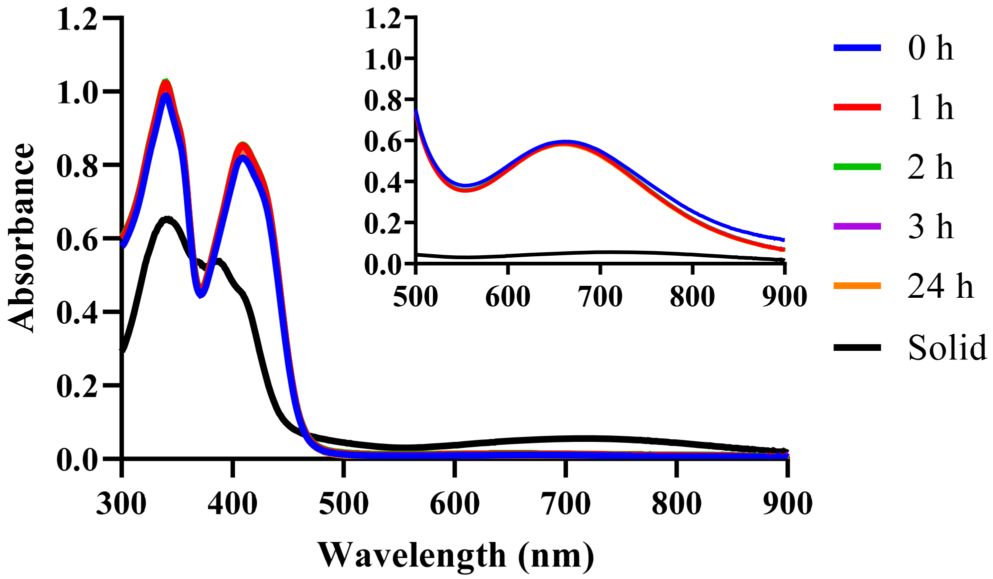

Figure S1. Electronic absorption spectra in UV–vis region of Cu_4L_4 in solid state (black line) and performed in DMSO at 1,25 × 10^{-5} mol L^{-1} from 0 to 24 h (color lines). Inset: expanded region between 500 to 900 nm of DMSO solution containing Cu_4L_4 at 1,25 × 10^{-3} mol L^{-1}.

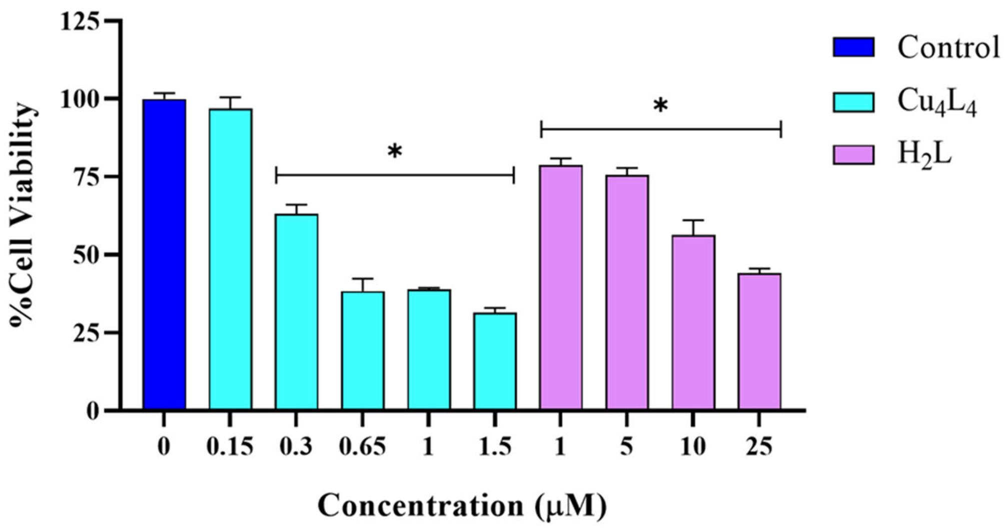

Figure S2. Cytotoxic effect of H_2L and Cu_4L_4 on MG-63 cells was evaluated by MTT assay. Cells were incubated with 0.5% DMSO in DMEM (Control) or with different concentrations of H_2L and Cu_4L_4 for 24 h. The results are expressed as the mean ± the standard error of the mean (SEM, n = 9). $^*p < 0.0001$ differences between control and treatment.

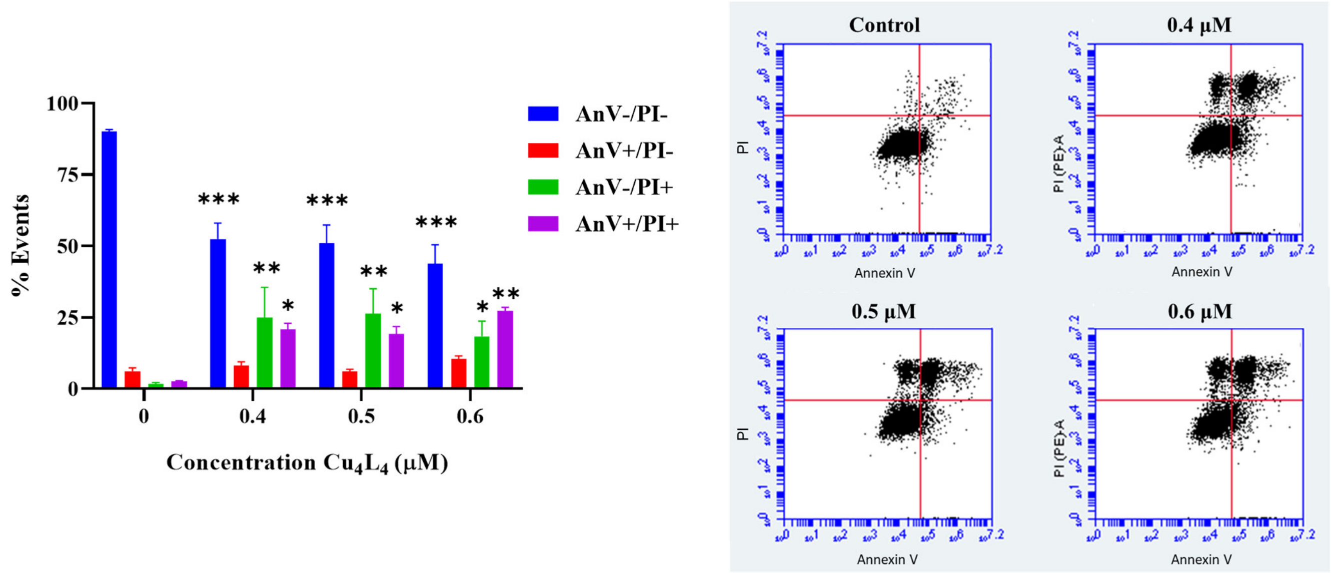

(**a**) (**b**)

Figure S3. Impact of Cu_4L_4 on MG-63 cell apoptosis induction. a) Cells were incubated in Dulbecco's modified Eagle's medium (DMEM) alone (Control) or with a variety of concentrations (0.4 to 0.6 μM) of Cu_4L_4 for 24 h. The results are expressed as the mean ± SEM. Results are representative of three independent experiments. $^*p < 0.05$, $^{**}p < 0.001$, $^{***}p < 0.0001$ differences between Control and treatment. b) Representative dot plots showing Annexin V-FITC/PI staining of apoptosis assay.

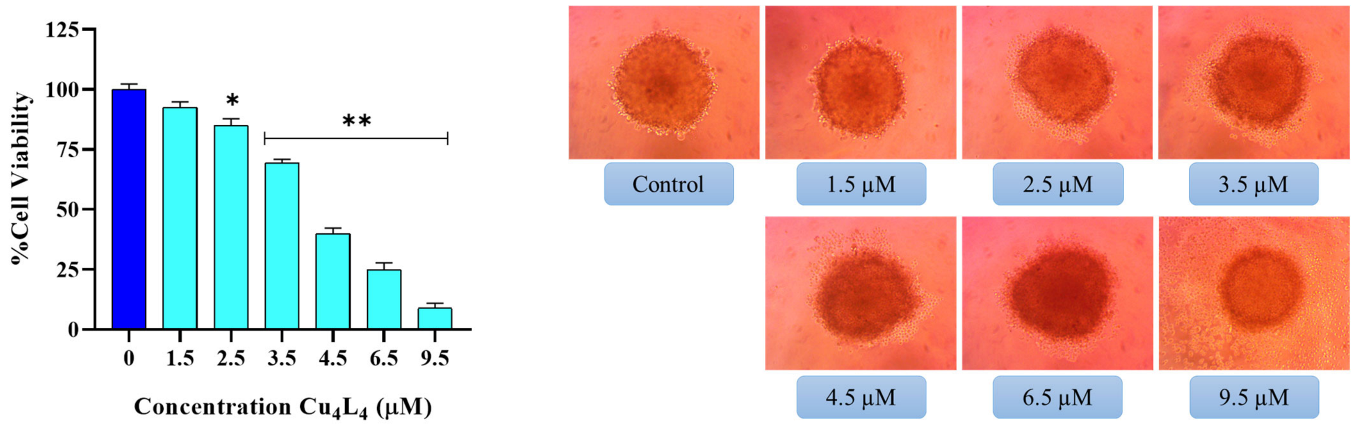

(**a**) (**b**)

Figure S4. Cell viability of the MG-63 spheroids evaluated with resazurin probe. a) percentage of cell viability of MCS treated with 0.5% DMSO in DMEM (Control) or different concentrations (1.5-9.5 µM) of Cu_4L_4 for 24 h. The results are expressed as the mean ± SEM (n = 12). **p* < 0.001 and ***p* < 0.0001 differences between Control and treatment. b) Representative images of the spheroids treated with the Control or complex (1.5-9.5 µM).

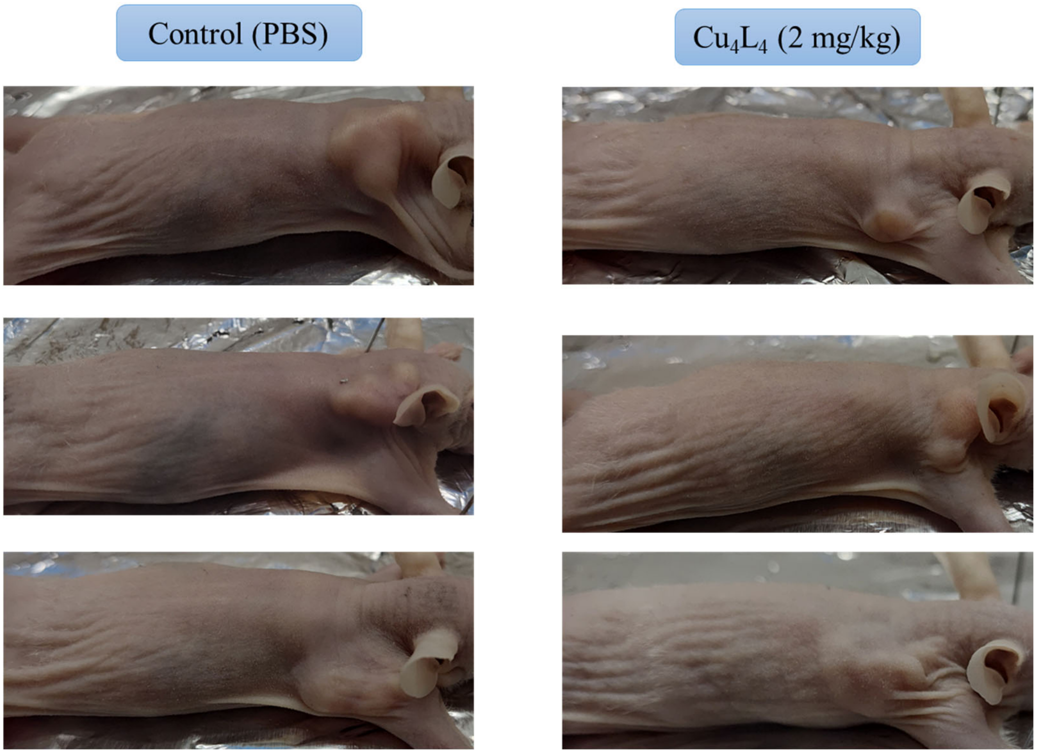

Figure S5. Representative photos of nude mice with OSA xenografts from the groups treated with Cu_4L_4 or Control (PBS) at day 45.

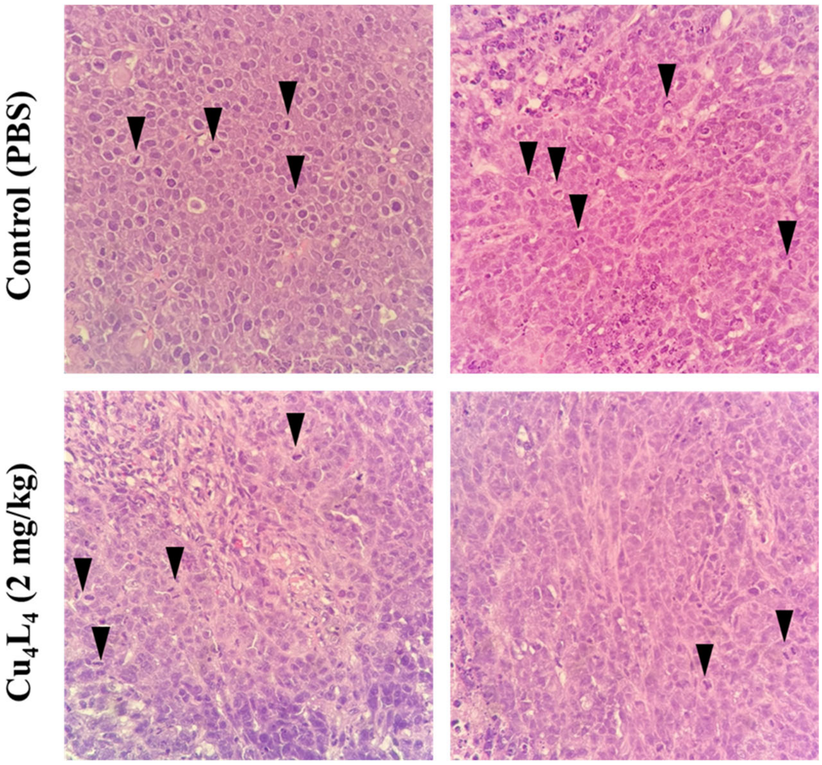

Figure S6. Representative images of H&E-stained tumor slides belonging to animals treated with PBS (Control) or Cu_4L_4. The arrows show tumor cell mitotic bodies (X400 magnification).

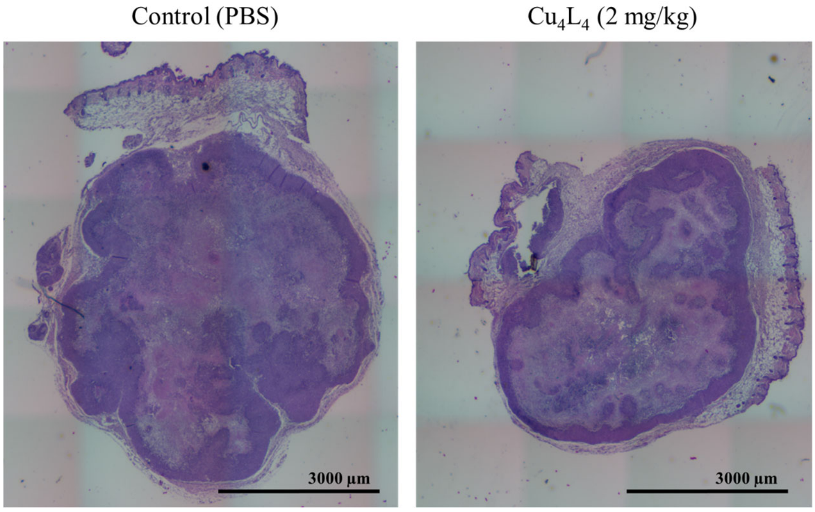

Figure S7. Representative digitally-stitched images of complete tumor sections from Control (left) and Cu_4L_4 (right) treatment groups. Scale bars = 3000 µm.

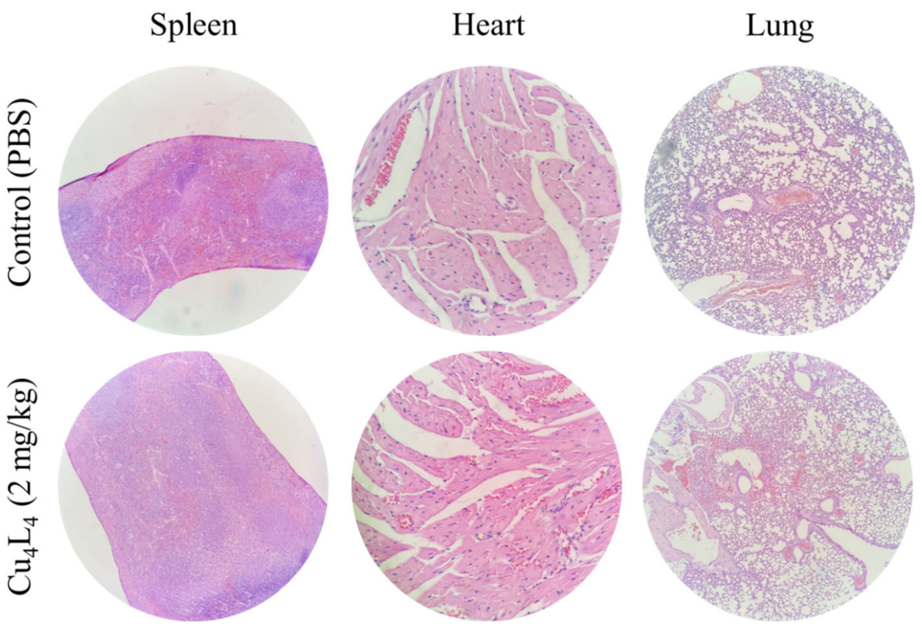

Figure S8. Representative images of H&E-stained spleen, heart and lung slides belonging to animals treated with PBS (Control) or Cu_4L_4. Images of spleen and lung were taken at X100 magnification, and heart with X400 magnification.

Table S1. Experimental electronic spectra of the complex in DMSO solution and in the solid state. Band maxima are given in nm. The molar absorptivity (in $M^{-1}cm^{-1}$) is in parentheses. The proposed assignment is also given.

**Experimental in DMSO (ε, $M^{-1}cm^{-1}$)**	**Experimental, Solid sample**	**Assignment**
696 (80)	721	d → d
431 (shoulder)	411 (shoulder)	d → d
409 (1.6 x 104)	387	LMCT
354 (shoulder)	341	Intra-ligand

Table S2. IC_{50} (μM) values of Cu_4L_4 and H_2L over MG-63 cells at 48 and 72 h of treatment.

**Compound**	**48 h**	**72 h**
Cu_4L_4	0.371 ± 0.003	0.363 ± 0.009
H_2L	4,785 ± 2,279	4,001 ± 0,871
